# Characterization of Dysregulated lncRNA-Associated ceRNA Network Reveals Novel lncRNAs With ceRNA Activity as Epigenetic Diagnostic Biomarkers for Osteoporosis Risk

**DOI:** 10.3389/fcell.2020.00184

**Published:** 2020-03-31

**Authors:** Meijie Zhang, Luyang Cheng, Yina Zhang

**Affiliations:** Department of Geriatrics, The Second Affiliated Hospital of Harbin Medical University, Harbin Medical University, Harbin, China

**Keywords:** competing endogenous RNAs, long non-coding RNAs, osteoporosis, biomark, epigenetics

## Abstract

The altered expression of long non-coding RNAs (lncRNAs) has been implicated in the development and human diseases. However, functional roles and regulatory mechanisms of lncRNA as competing endogenous RNAs (ceRNAs) in osteoporosis and their potential clinical implication for osteoporosis risk are largely unexplored. In this study, we performed integrated analysis for paired expression profiles and regulatory relationships of dysregulated lncRNAs, mRNAs, and miRNAs based on “ceRNA hypothesis,” and constructed an osteoporosis-related dysregulated miRNA-mediated lncRNA–mRNA ceRNA network (DysCeNet) composed of 105 nodes (including eight miRNAs, 24 mRNAs, and 73 lncRNAs) and 515 edges. Functional analysis suggested that the DysCeNet was involved in known osteoporosis or bone metabolism-related biological processes and pathways. Then, we performed random forest-based feature selection for 73 lncRNAs with ceRNA activity and identified 25 of 73 lncRNAs as potential diagnostic biomarkers. A random forest-based classifier composed of 25 lncRNA biomarkers (RF-25lncRNA) was developed for predicting osteoporosis risk. Performance evaluation with the leave-one-out cross-validation (LOOCV) procedure showed that the RF-25lncRNA achieved a good performance in distinguishing high- and low-bone mineral density (BMD) subjects in different osteoporosis datasets. Our study for the first time revealed a global view of lncRNA-associated ceRNA regulation in osteoporosis and provided novel lncRNAs with ceRNA activity as candidate epigenetic diagnostic biomarkers for early detection of osteoporosis risk.

## Introduction

Osteoporosis is a progressive systemic skeletal disease with low bone density and deterioration of bone architecture (Garnero, 2017; Tu et al., 2018). It is estimated that over 14 million men and women in the United States will have osteoporosis by 2020 (Burge et al., 2007). People with osteoporosis substantially have an increased risk of bone fragility and fracture, leading to increase pain, disability, nursing home placement, total health care costs, and mortality (Tu et al., 2018). Early detection and intervention for osteoporosis risk are an effective way to delay the development of disease and improve the quality of life of patients. Therefore, there is an urgent need to identify novel molecular biomarkers in the clinical assessment of osteoporosis risk.

With the development and application of high-throughput omics technologies, it has been shown that osteoporosis has genetic and molecular heterogeneity like many other common complex diseases, and disruption in some molecular pathways can disturb the equilibrium of bone turnover and thereby contribute to osteoporosis (Al Anouti et al., 2019). Long non-coding RNAs (lncRNAs) are a newly discovered class of non-coding RNAs (ncRNAs) that are longer than 200 bp (Pelechano and Steinmetz, 2013) and have attracted much attention in recent medical studies. A large number of studies have demonstrated that lncRNAs is an important player of the genomic regulatory network and is involved in a wide variety of biological progress (Mercer and Mattick, 2013; Guo et al., 2016; Quinn and Chang, 2016; Bunch, 2018; Sun et al., 2020). lncRNAs have been attributed to various functions in development, differentiation, and human disease by negatively or positively regulating gene expression at the transcriptional, post-transcriptional, and epigenetic levels (Kornienko et al., 2013; Maass et al., 2014). Growing functional roles of lncRNAs has been highlighted in osteoporosis during recent studies. For example, *XIST*, a well-known major effector of the X-inactivation process, was recently reported to promote osteoporosis through inhibiting bone marrow mesenchymal stem cell differentiation (Chen et al., 2019). Mei et al. (2019) found that lncRNA *ZBTB40-IT1* played a critical role in bone metabolism and can be modulated by osteoporosis GWAS risk SNPs suppresses osteogenesis. It has become increasingly clear that lncRNAs can act as competing endogenous RNAs (ceRNAs) to interact other RNA molecules by competing for binding to shared microRNAs, which has been implicated in development and human disease including osteogenesis (Tay et al., 2014; Huang et al., 2019a, b; Silva et al., 2019). However, genome-wide exploration for miRNA-mediated lncRNA-associated ceRNA mechanism in osteoporosis and their potential clinical implication for osteoporosis risk remained largely unknown.

In this study, we tried to construct a global miRNA-mediated lncRNA–mRNA ceRNA network in the development of osteoporosis by integrating paired expression profiles and regulatory relationship of lncRNAs, mRNAs, and miRNAs based on “ceRNA hypothesis,” and further to uncover novel lncRNAs with ceRNA activity as epigenetic diagnostic biomarkers for identifying people at high risk for developing osteoporosis.

## Materials and Methods

### Patients and Samples

Ten samples [including five high-bone mineral density (BMD) subjects and five low-BMD subjects] with corresponding transcriptome gene expression microarray data (Affymetrix Human Exon 1.0 ST Array) and epigenomic miRNA microarray data (Affymetrix Multispecies miRNA-2 Array) were obtained from the Gene Expression Omnibus (GEO) database (the accession number is GSE62589^1^). Two other osteoporosis datasets with transcriptome gene expression microarray data were obtained from the GEO database, including GSE56814 dataset (16 high-BMD subjects and 15 low-BMD subjects) (the accession number is GSE56814^2^) and GSE13850 dataset (10 high-BMD subjects and 10 low-BMD subjects) (the accession number is GSE13850^3^).

### Acquisition and Analysis of Expression Profiles

Raw transcriptome gene expression microarray data (CEL files) profiled on Affymetrix GeneChip Human Exon 1.0 ST Array (HuEx-1_0-st) and Affymetrix Human Genome U133A Array (HG-U133A) were obtained from the GSE63402 and GSE13850. These raw data were processed and normalized using the Robust Multichip Average (RMA) algorithm of R package “oligo” for background subtraction, quantile normalization, and summarization. Then, all probes of microarray were mapped into protein-coding genes using the R package “biomaRt” (Durinck et al., 2009). LncRNA expression profiles were obtained using repurposing strategy by mapping array probes into the human genome (GRCh 38) and lncRNA annotations from the GENCODE database^4^ (Zhou et al., 2016) using SeqMap tool (Jiang and Wong, 2008). Human mature miRNAs were retrieved from the miRNA microarray. Finally, expression profiles of 20,068 mRNA, 7821 lncRNAs, and 1100 miRNA were obtained for further analysis.

Differential expression analysis of lncRNAs, miRNAs, and mRNAs between high- and low-BMD subjects was performed using the R package “limma” (Ritchie et al., 2015). Those lncRNAs, miRNAs, and mRNAs with *p* < 0.05 were considered as differentially expressed genes. Hierarchical clustering was performed to investigate the expression patterns between high- and low-BMD subjects.

### Construction of Dysregulated lncRNA-Associated ceRNA Network

The experimentally validated miRNA–mRNA and miRNA–lncRNA interaction data were collected from the TarBase database^5^ (Li et al., 2014). The dysregulated lncRNA-associated ceRNA network (DysCeNet) in osteoporosis was constructed based on the “ceRNA hypothesis” as follows: (i) Pearson correlation coefficient (PCC) was calculated to measure the expression correlation between differentially expressed mRNAs and lncRNAs from matched mRNA and lncRNA expression profiles. Those dysregulated lncRNA–mRNA pairs with PCC > 0.5 were considered as candidate co-dysregulated lncRNA–mRNA ceRNA crosstalk; (ii) expression correlation between differentially expressed miRNAs and differentially expressed mRNAs, and between differentially expressed miRNAs and differentially expressed lncRNAs was evaluated using PCC; (iii) for a candidate co-dysregulated lncRNA–mRNA ceRNA crosstalk, both mRNAs and lncRNAs in this lncRNA–mRNA ceRNA crosstalk are targeted and co-expressed negatively with a common miRNA; this candidate co-dysregulated lncRNA–mRNA ceRNA crosstalk was selected as dysregulated miRNA-mediated lncRNA–mRNA ceRNA crosstalk; (iv) all miRNA-mediated lncRNA–mRNA ceRNA crosstalks were integrated to form a global DysCeNet.

### Identification of lncRNA Biomarkers With ceRNA Activity Using a Machine Learning Method

To identify potential lncRNA biomarkers with ceRNA activity, a random forest approach and leave-one-out cross-validation (LOOCV) were used to select optimal lncRNAs biomarkers using the R package “randomForest” and out-of-bag (OOB) error, which measure the performance of the model on the training set (Lv et al., 2019; Tan et al., 2019). The OOB error will produce an unbiased estimate for the classification error, while the bagging method will decrease the chance of overfitting (Toth et al., 2019). Then, a random forest-based classifier was built using the optimal lncRNA biomarkers, and a receiver operating characteristic (ROC) curve and the area under ROC curve (AUC) was used to measure the diagnostic performance of the lncRNA classifier (Lai et al., 2019).

### Functional Enrichment Analysis

Functional enrichment analysis of Gene Ontology (GO) and Kyoto Encyclopedia of Genes and Genomes (KEGG) for mRNAs in the DysCeNet was conducted to predict potential biological functions of lncRNAs in the DysCeNet using the R package “clusterprofiler” (Yu et al., 2012). Those significantly enriched GO terms with *p* < 0.05 with mutually overlapping gene sets were clustered together using the Enrichment Map plugin in Cytoscape environment (Merico et al., 2010).

## Results

### Identification of Differentially Expressed mRNAs, miRNAs, and lncRNAs Associated With Osteoporosis

**FIGURE 1:**
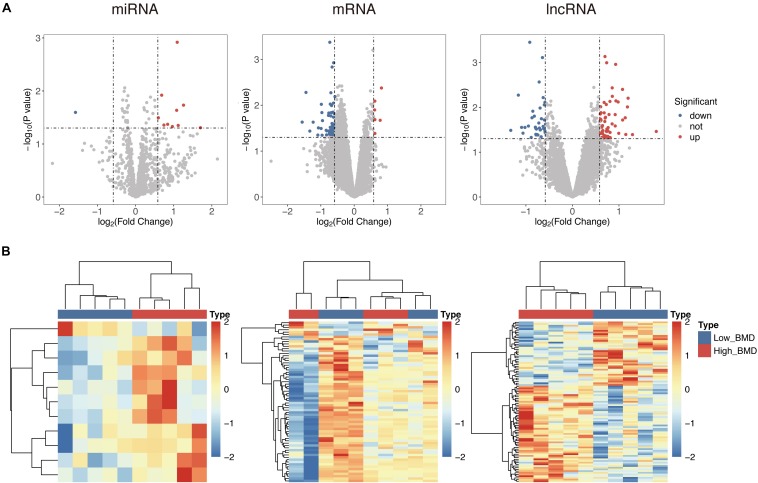
Differential expressed analysis between high- and low-BMD subjects. **(A)** Volcano plot of the distribution of differentially expressed mRNAs, miRNAs, and lncRNAs. **(B)** Hierarchical clustering heatmap and dendrogram of 10 samples based on differentially expressed mRNAs, miRNAs, and lncRNAs.

To identify potential risk mRNAs, miRNAs, and lncRNAs in osteoporosis, we performed a comparative analysis for expression profiles of mRNAs, miRNAs, and lncRNAs between high- and low-BMD subjects. A total of 68 mRNAs, 11 miRNAs, and 95 lncRNAs were identified as differentially expressed (*p* < 0.05) in high-BMD subjects compared with low-BMD subjects (Supplementary Table S1). Among them, there were 73 unregulated genes (including six mRNAs, 10 miRNAs, and 57 lncRNAs) and 101 downregulated genes (including 62 mRNAs, one miRNA, and 38 lncRNAs) in high-BMD subjects compared with low-BMD subjects (Figure 1A). Hierarchical clustering analysis showed that expression patterns of these differentially expressed mRNAs, miRNAs, and lncRNAs were capable of distinguishing high-BMD subjects from low-BMD subjects (Figure 1B).

### Construction and Analysis of Dysregulated lncRNA-Associated ceRNA Network

**FIGURE 2:**
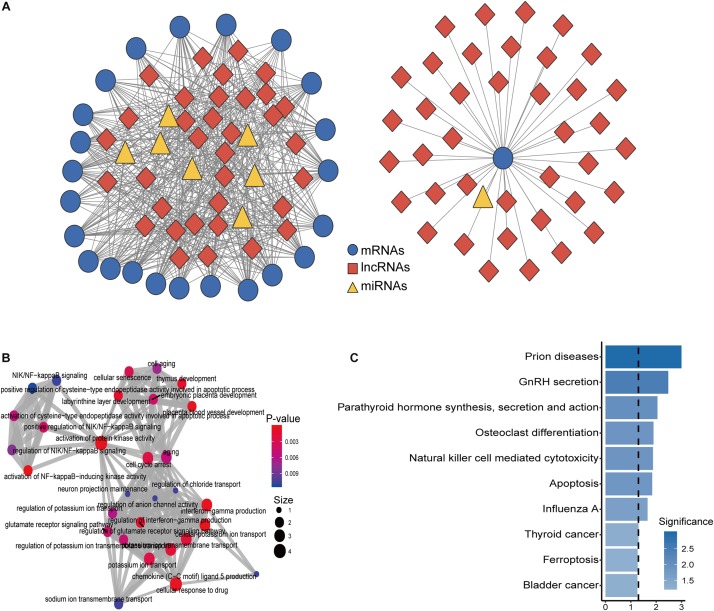
Construction and characterization of dysregulated lncRNA-associated ceRNA network (DysCeNet). **(A)** A global view of the DysCeNet in osteoporosis. **(B)** The functional enrichment map of GO terms for mRNAs as ceRNA counterparts of lncRNA biomarkers. **(C)** Enriched KEGG pathways for mRNAs as ceRNA counterparts of lncRNA biomarkers.

To construct an osteoporosis-related dysregulated miRNA-mediated lncRNA–mRNA ceRNA network, we performed an integrated analysis for paired expression profiles and regulatory relationships of dysregulated lncRNAs, mRNAs, and miRNAs of 10 samples in the GSE62589 as described in Section “Materials and Methods.” Finally, an osteoporosis-related dysregulated miRNA-mediated lncRNA–mRNA ceRNA network was constructed and was composed of 105 nodes and 515 edges (including eight miRNAs, 24 mRNAs, and 73 lncRNAs) (Figure 2A) (Supplementary Table S2). To further explore the functional implication of the DysCeNet, we performed functional enrichment analysis for mRNAs in the DysCeNet and found that mRNAs in the DysCeNet were significantly enriched in blood vessel development, NIK/NF-kappaB signaling, bone mineralization involved in bone maturation, osteoclast differentiation, and cell aging.

### Identification of Potential lncRNA Biomarkers for the Osteoporosis Risk

**FIGURE 3:**
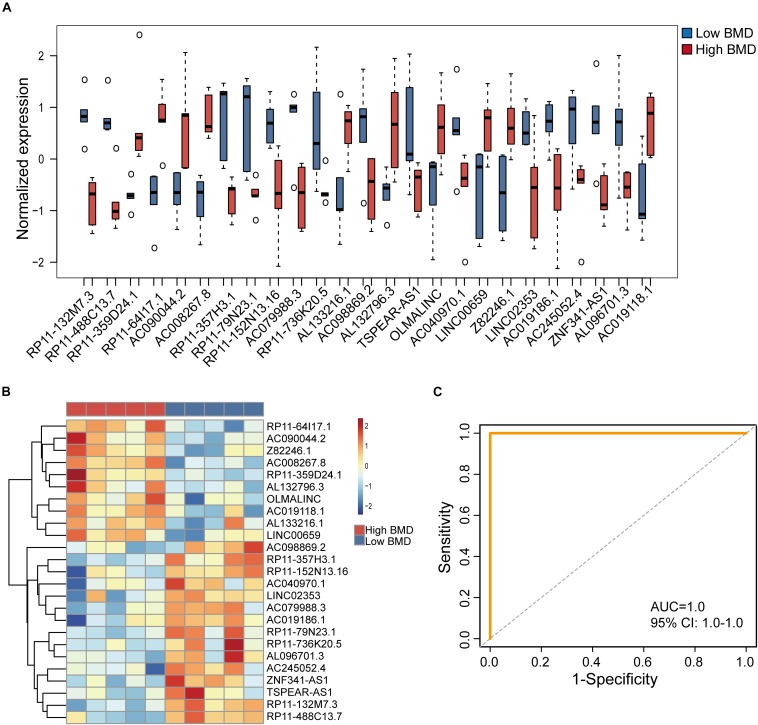
Performance evaluation of identified 25 lncRNA biomarkers in the discovery dataset. **(A)** Expression pattern of 2525 lncRNA biomarkers in high-BMD and low-BMD subjects. **(B)** Hierarchical clustering heatmap and dendrogram of 10 samples based on expression patterns of 25 lncRNA biomarkers. **(C)** The ROC curves of RF-25lncRNA in the GSE62589 dataset.

To identify potential lncRNA biomarkers for the osteoporosis risk, we performed feature selection for lncRNAs in the DysCeNet using a random forest model. Finally, 25 lncRNAs of 73 lncRNAs in the DysCeNet were identified as potential biomarkers for the osteoporosis risk according to their discriminative power using OOB error (Table 1). Of them, 10 lncRNA biomarkers are up-regulated and 15 lncRNA biomarkers are down-regulated in high-BMD subjects compared with low-BMD subjects (Figure 3A). To test whether these 25 lncRNA biomarkers could efficiently distinguish high- and low-BMD subjects, we performed hierarchical clustering for 10 samples in the GSE62589 according to the expression pattern of 25 lncRNA biomarkers. As shown in Figure 3B, all 10 samples were classified into two clusters according to the expression pattern of seven lncRNA biomarkers with 100% accuracy. The results of hierarchical clustering demonstrated the potential of 25 lncRNAs as diagnostic biomarkers for osteoporosis risk. Therefore, a random forest-based classifier composed of 25 lncRNA biomarkers was developed. The performance of the RF-25lncRNA for predicting osteoporosis risk was evaluated in the GSE62589 dataset using the LOOCV procedure, in which nine samples were used as the training set and the remaining one was served as the test sample. Results of performance evaluation showed that the RF-25lncRNA achieves a perfect predictive performance in distinguishing high- and low-BMD subjects with an AUC of 1.0 (Figure 3C).

**TABLE 1:** Genomic information of 25 lncRNA biomarkers with ceRNA activity for osteoporosis risk.

Ensembl id	Gene name	Location	*p*-value^a^
ENSG00000228290	RP11-132M7.3	Chr 6: 85,399,143-85,419,252 (+)	0.0008
ENSG00000258610	RP11-488C13.7	Chr 14: 77,245,295-77,248,592 (+)	0.0004
ENSG00000242009	RP11-359D24.1	Chr 3: 81,889,698-81,972,012 (+)	0.0078
ENSG00000254562	RP11-64I17.1	Chr 11: 38,668,026-38,695,791 (+)	0.0011
ENSG00000224239	AC090044.2	Chr 3: 898,807-899,774 (+)	0.0063
ENSG00000279785	AC008267.8	Chr 7: 66,474,556-66,475,749 (+)	0.0007
ENSG00000264108	RP11-357H3.1	Chr 18: 73,720,073-73,727,816 (−)	0.0027
ENSG00000227885	RP11-79N23.1	Chr 6: 53,605,547-53,611,820 (−)	0.0088
ENSG00000272599	RP11-152N13.16	Chr 10: 74,884,331-74,885,163 (−)	0.0053
ENSG00000236145	AC079988.3	Chr 2: 121,935,903-121,940,156 (+)	0.0061
ENSG00000255471	RP11-736K20.5	Chr 11: 86,603,256-86,636,079 (−)	0.0296
ENSG00000203496	AL133216.1	Chr 10: 38,453,181-38,466,176 (+)	0.0444
ENSG00000250893	AC098869.2	Chr 4: 40,426,119-40,427,585 (+)	0.0299
ENSG00000278990	AL132796.3	Chr 14: 98,916,902-98,917,801 (−)	0.0098
ENSG00000235890	TSPEAR-AS1	Chr 21: 44,506,807-44,516,575 (+)	0.0414
ENSG00000235823	OLMALINC	Chr 10: 100,373,099-100,454,043 (+)	0.0207
ENSG00000253210	AC040970.1	Chr 8: 141,124,717-141,130,064 (−)	0.0281
ENSG00000228705	LINC00659	Chr 20: 62,774,121-62,776,996 (−)	0.0193
ENSG00000236054	Z82246.1	Chr 22: 32,583,300-32,584,204 (+)	0.0097
ENSG00000251329	LINC02353	Chr 4: 32,351,038-32,353,220 (+)	0.0171
ENSG00000234584	AC019186.1	Chr 2: 157,725,708-157,736,005 (−)	0.0104
ENSG00000237017	AC245052.4	Chr 19: 54,119,511-54,125,343 (−)	0.0191
ENSG00000230753	ZNF341-AS1	Chr 20: 33,787,373-33,811,109 (−)	0.0077
ENSG00000240591	AL096701.3	Chr 22: 31,452,881-31,464,304 (+)	0.0179
ENSG00000226649	AC019118.1	Chr 2: 3,156,756-3,157,797 (+)	0.0087

*^*a*^*p*-value was obtained from differential expression analysis.*

### Further Validation of lncRNA Biomarkers for the Osteoporosis Risk in Two Other Independent Datasets

**FIGURE 4:**
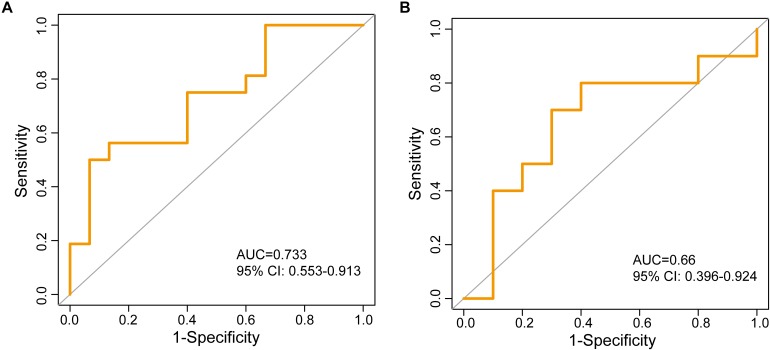
Independent validation of identified 25 lncRNA biomarkers in two independent testing datasets. **(A)** The ROC curves of RF-25lncRNA in the GSE56814 dataset. **(B)** The ROC curves of two lncRNA biomarkers (*RP11-488C13.7* and *RP11-152N13.16*) in the GSE13850 dataset.

To test the robustness of the lncRNA biomarkers for the osteoporosis risk, 25 lncRNA biomarkers were applied to the independent GSE56814 dataset. The RF-25lncRNA correctly classified 11 of 15 low-BMD subjects and nine of 16 high-BMD subjects, achieving an AUC of 0.733 (95% CI: 0.553–0.913) with an accuracy of 64.5% (Figure 4A).

The performance of the lncRNA biomarkers was further tested in another independent GSE13850 dataset. However, transcriptome gene expression data of the GSE13850 was profiled on the HG-U133A platform, and only two lncRNAs (*RP11-488C13.7* and *RP11-152N13.16*) of 25 lncRNA biomarkers in the RF-25lncRNA were covered on the HG-U133A platform. When two lncRNA biomarkers (*RP11-488C13.7* and *RP11-152N13.16*) were applied to the 20 samples of the GSE13850, two lncRNA biomarkers (*RP11-488C13.7* and *RP11-152N13.16*) correctly classified seven of 10 low-BMD subjects and seven of 10 high-BMD subjects, achieving an AUC of 0.66 (95% CI: 0.396–0.924) with an accuracy of 70% (Figure 4B).

## Discussion

Early detection and intervention for osteoporosis are crucial to prevent fragility fractures and delay disease progression. Traditional markers for osteoporosis risk are BMD, vitamin D, alkaline phosphatase, and so on (Parveen et al., 2019). Increasing evidence has suggested that altered molecular profiles contributed to the osteoporosis and outcome, which provided novel insights into molecular basis of osteoporosis and also highlighted the potential of molecular factors as markers for osteoporosis diagnosis and prognosis (Yu et al., 2013; Makitie et al., 2018; Gong et al., 2019; Ukon et al., 2019). Biomarker identification has been proven to be an effective way to recognize people at high risk for developing osteoporosis and have attracted much attention in the clinical decision-making for osteoporosis management. Previous studies have focused on mRNA profiles and miRNA profiles and identified some candidate mRNA- or miRNA-based biomarkers. For example, circulating miRNAs, *hsa-miR-122-5p* and *hsa-miR-4516*, have been found to be diagnostic biomarkers for osteoporosis risk (Mandourah et al., 2018). Another study by Seeliger et al. (2014) also identified five freely circulating miRNAs associated with osteoporosis fractures. There is increasing evidence that lncRNAs also play important roles in the pathogenesis of osteoporosis (Peng et al., 2018; Feng et al., 2019; Liu et al., 2019). In this study, we obtained lncRNA profiles of osteoporosis patients by repurposing array probes on publicly available microarray data and performed genome-wide analysis for expression patterns of lncRNAs, miRNAs, and mRNAs between high- and low-BMD subjects. A total of 68 mRNAs, 11 miRNAs, and 95 lncRNAs were found to be associated with osteoporosis, which provided a candidate resource for experimental scientist for studying the molecular mechanism of osteoporosis.

Through many candidate osteoporosis-related mRNAs, miRNAs, and lncRNAs were identified in our study, regulatory relationships and mechanisms among these different types of RNA molecules in the development of osteoporosis are still unknown. It is well known that different RNA molecules can act as ceRNAs to communicate with and co-regulate each other by competing for binding to shared miRNAs (Tay et al., 2014). LncRNAs have been reported as key components of the ceRNA-mediated regulatory network, and aberrant miRNA-mediated lncRNA–mRNA ceRNA crosstalk has been implicated in many human complex diseases, including cancers. However, functional roles and regulatory mechanisms of lncRNA as ceRNAs in the development of osteoporosis and their potential implication for osteoporosis are largely unexplored. To explore the ceRNA activity of lncRNAs in the development of osteoporosis, we performed integrated analysis for paired expression profiles of dysregulated 68 mRNAs, 11 miRNAs, and 95 lncRNAs and miRNA–target regulatory relationship based on “ceRNA hypothesis,” and constructed an osteoporosis-related miRNA-mediated dysregulated lncRNA–mRNA ceRNA network (DysCeNet) composed of 105 nodes and 515 edges (including eight miRNAs, 24 mRNAs, and 73 lncRNAs). Network analysis suggested that a large proportion of deregulated lncRNAs (76.8%, 73/95) in osteoporosis function as ceRNA and communicated with 24 mRNAs by competing for eight common miRNAs (Figure 2A), which implied that extensive variation in miRNAs and lncRNAs disrupted the miRNA-mediated lncRNA–mRNA ceRNA regulatory network contributing to osteoporosis at the post-transcriptional level. Functional analysis through functional enrichment analysis for mRNAs in the DysCeNet found that mRNAs as ceRNA counterparts of lncRNA biomarkers were involved in known osteoporosis or bone metabolism-related biological progression and pathways, including blood vessel development, NIK/NF-kappaB signaling, bone mineralization involved in bone maturation, osteoclast differentiation, and cell aging (Figures 2B,C). For example, blood vessels in the bone play vital roles for the formation of new bone and promoting blood vessel growth could reverse the weakening of bones and treat osteoporosis (Sivaraj and Adams, 2016). NIK/NF-kappaB signaling has been shown to play an important role in the positive and negative regulation of cytokine-mediated osteoclast formation and activation (Boyce et al., 2015). These results suggested that dysregulated expression of lncRNA ceRNAs and the resultant perturbation in miRNA-mediated lncRNA–mRNA crosstalk in the DysCeNet are involved in osteoporosis-biological processes and contributed to the osteoporosis.

A large number of studies have indicated the superior potential of lncRNAs as diagnostic and prognostic biomarkers compared with protein-coding genes due to the fact that lncRNAs were expressed in much more cell- type-, tissue-, and disease-specific patterns that are closely more associated with their function (Hauptman and Glavaè, 2013; Chen et al., 2018). lncRNA biomarkers have been widely investigated and identified in various cancers during the past years (Zhou et al., 2018a, b, 2019; Bao et al., 2019). To explore the potential application of lncRNAs with ceRNA activity as diagnostic biomarkers for osteoporosis risk, we performed random forest-based feature selection for 73 lncRNAs with ceRNA activity and identified 25 of 73 lncRNAs as potential diagnostic biomarkers. To accelerate the clinical application, we also developed a random forest-based classifier (RF-25lncRNA) composed of seven lncRNA biomarkers and test the performance of the RF-25lncRNA in different osteoporosis datasets. Performance evaluation of the LOOCV procedure showed that the RF-25lncRNA achieved a good performance in distinguishing high- and low-BMD subjects in three osteoporosis datasets. These results demonstrated that seven lncRNA with ceRNA activity may become reliable and powerful epigenetic diagnostic biomarkers for early detection of osteoporosis risk.

There are some limitations to this study. First, the performance of newly identified lncRNA biomarkers was validated only in three osteoporosis datasets because of no other publicly available osteoporosis datasets. Second, the biological functions of newly identified lncRNA biomarkers are unknown, although they were found to have ceRNA activity in our study. Therefore, more laboratory and clinical researches were needed.

## Data Availability Statement

Publicly available datasets were analyzed in this study. This data can be found here: Three osteoporosis datasets were obtained from the Gene Expression Omnibus (GEO) database (GSE62589, https://www.ncbi.nlm.nih.gov/geo/query/acc.cgi?acc=GSE62589, GSE56814, https://www.ncbi.nlm.nih.gov/geo/query/acc.cgi?acc=GSE56814, and GSE13850, https://www.ncbi.nlm.nih.gov/geo/query/acc.cgi?acc=GSE13850).

## Author Contributions

YZ conceived and designed the experiments. MZ and LC analyzed the data. MZ wrote the manuscript. All authors read and approved the final manuscript.

## Conflict of Interest

The authors declare that the research was conducted in the absence of any commercial or financial relationships that could be construed as a potential conflict of interest.

## Footnotes

1 https://www.ncbi.nlm.nih.gov/geo/query/acc.cgi?acc=GSE62589

2 https://www.ncbi.nlm.nih.gov/geo/query/acc.cgi?acc=GSE56814

3 https://www.ncbi.nlm.nih.gov/geo/query/acc.cgi?acc=GSE13850

4 https://www.gencodegenes.org/

5 http://starbase.sysu.edu.cn/

## Supplementary Material

TABLE S1 List of differentially expressed miRNAs, mRNAs and lncRNAs between high-BMD subjects and low-BMD subjects.

TABLE S2 An osteoporosis-related dysregulated miRNA-mediated lncRNA-mRNA ceRNA network.

## References

[B1] Al Anouti F. Taha Z. Shamim S. Khalaf K. Al Kaabi L. Alsafar H. J. (2019). An insight into the paradigms of osteoporosis: from genetics to biomechanics. *Bone Rep.* 11:100216. 10.1016/j.bonr.2019.100216 31372373 PMC6661363

[B2] Bao S. Zhao H. Yuan J. Fan D. Zhang Z. Su J. (2019). Computational identification of mutator-derived lncRNA signatures of genome instability for improving the clinical outcome of cancers: a case study in breast cancer. *Brief Bioinform.* [Epub ahead of print] 31665214 10.1093/bib/bbz118

[B3] Boyce B. F. Xiu Y. Li J. Xing L. Yao Z. J. E. (2015). NF-(B-mediated regulation of osteoclastogenesis. *Endocrinol. Metab.* 30 35–44. 10.3803/EnM.2015.30.1.35 PMC4384681 25827455

[B4] Bunch H. (2018). Gene regulation of mammalian long non-coding RNA. *Mol. Genet. Genomics* 293 1–15. 10.1007/s00438-017-1370-9 28894972

[B5] Burge R. Dawson-Hughes B. Solomon D. H. Wong J. B. King A. Tosteson A. (2007). Incidence and economic burden of osteoporosis-related fractures in the United States, 2005-2025. *J. Bone. Miner. Res.* 22 465–475. 10.1359/jbmr.061113 17144789

[B6] Chen L. Zhang Y.-H. Pan X. Liu M. Wang S. Huang T. (2018). Tissue expression difference between mRNAs and lncRNAs. *Eur. Rev. Med. Pharmacol. Sci.* 19:3416. 10.3390/ijms19113416 30384456 PMC6274976

[B7] Chen X. Yang L. Ge D. Wang W. Yin Z. Yan J. (2019). Long non-coding RNA XIST promotes osteoporosis through inhibiting bone marrow mesenchymal stem cell differentiation. *Exp. Ther. Med.* 17 803–811. 10.3892/etm.2018.7033 30651866 PMC6307375

[B8] Durinck S. Spellman P. T. Birney E. Huber W. (2009). Mapping identifiers for the integration of genomic datasets with the R/Bioconductor package biomaRt. *Nat. Protoc.* 4 1184–1191. 10.1038/nprot.2009.97 19617889 PMC3159387

[B9] Feng J. Wang J. Li C. H. (2019). LncRNA GAS5 overexpression alleviates the development of osteoporosis through promoting osteogenic differentiation of MSCs via targeting microRNA-498 to regulate RUNX2. *Eur. Rev. Med. Pharmacol. Sci.* 23 7757–7765. 10.26355/eurrev_201909_18985 31599401

[B10] Garnero P. (2017). The utility of biomarkers in osteoporosis management. *Mol. Diagn. Ther.* 21 401–418. 10.1007/s40291-017-0272-1 28271451

[B11] Gong R. Ren S. Chen M. Wang Y. Zhang G. Shi L. (2019). Bioinformatics analysis reveals the altered gene expression of patients with postmenopausal osteoporosis using liuweidihuang pills treatment. *Biomed. Res. Int.* 2019:1907906. 10.1155/2019/1907906 30809532 PMC6369488

[B12] Guo X. Gao L. Wang Y. Chiu D. K. Wang T. Deng Y. (2016). Advances in long non-coding RNAs: identification, structure prediction and function annotation. *Brief Funct. Genomics* 15 38–46. 10.1093/bfgp/elv022 26072035 PMC5863772

[B13] Hauptman N. Glavaè D. (2013). Long non-coding RNA in cancer. *Noncoding RNA Res.* 14 4655–4669. 10.3390/ijms14034655 PMC3634483 23443164

[B14] Huang R. Wu J. Zheng Z. Wang G. Song D. Yan P. (2019a). The construction and analysis of ceRNA network and Patterns of immune infiltration in Mesothelioma with bone metastasis. *Front. Bioeng. Biotechnol.* 7:257. 10.3389/fbioe.2019.00257 31681739 PMC6813567

[B15] Huang R. Zeng Z. Li G. Song D. Yan P. Yin H. (2019b). The construction and comprehensive analysis of ceRNA networks and tumor-infiltrating immune cells in bone metastatic melanoma. *Front. Genet.* 10:828. 10.3389/fgene.2019.00828 31608101 PMC6774271

[B16] Jiang H. Wong W. H. (2008). SeqMap: mapping massive amount of oligonucleotides to the genome. *Bioinformatics* 24 2395–2396. 10.1093/bioinformatics/btn429 18697769 PMC2562015

[B17] Kornienko A. E. Guenzl P. M. Barlow D. P. Pauler F. M. (2013). Gene regulation by the act of long non-coding RNA transcription. *BMC Biol.* 11:59. 10.1186/1741-7007-11-59 23721193 PMC3668284

[B18] Lai H.-Y. Zhang Z.-Y. Su Z.-D. Su W. Ding H. Chen W. (2019). iProEP: a computational predictor for predicting promoter. *Mol. Ther. Nucleic Acids* 17 337–346. 10.1016/j.omtn.2019.05.028 31299595 PMC6616480

[B19] Li J. H. Liu S. Zhou H. Qu L. H. Yang J. H. (2014). starBase v2.0: decoding miRNA-ceRNA, miRNA-ncRNA and protein-RNA interaction networks from large-scale CLIP-Seq data. *Nucleic Acids Res.* 42 D92–D97. 10.1093/nar/gkt1248 24297251 PMC3964941

[B20] Liu S. Huang H. Chai S. Wei H. Huang J. Wan L. (2019). Expression profile analysis of long non-coding RNA in skeletal muscle of osteoporosis by microarray and bioinformatics. *J. Biol. Eng.* 13:50. 10.1186/s13036-019-0180-5 31164921 PMC6544974

[B21] Lv H. Zhang Z.-M. Li S.-H. Tan J.-X. Chen W. Lin H. (2019). Evaluation of different computational methods on 5-methylcytosine sites identification. *Brief Bioinform.* [Epub ahead of print], 31157855 10.1093/bib/bbz048

[B22] Maass P. G. Luft F. C. Bähring S. (2014). Long non-coding RNA in health and disease. *J. Mol. Med.* 92 337–346. 24531795 10.1007/s00109-014-1131-8

[B23] Makitie R. E. Hackl M. Niinimaki R. Kakko S. Grillari J. Makitie O. (2018). Altered MicroRNA profile in osteoporosis caused by impaired WNT signaling. *J. Clin. Endocrinol. Metab.* 103 1985–1996. 10.1210/jc.2017-02585 29506076

[B24] Mandourah A. Y. Ranganath L. Barraclough R. Vinjamuri S. Hof R. V. T. Hamill S. (2018). Circulating microRNAs as potential diagnostic biomarkers for osteoporosis. *Sci. Rep.* 8 1–10. 29849050 10.1038/s41598-018-26525-y PMC5976644

[B25] Mei B. Wang Y. Ye W. Huang H. Zhou Q. Chen Y. (2019). LncRNA ZBTB40-IT1 modulated by osteoporosis GWAS risk SNPs suppresses osteogenesis. *Biol. Med.* 138 151–166. 10.1007/s00439-019-01969-y 30661131

[B26] Mercer T. R. Mattick J. S. (2013). Structure and function of long non-coding RNAs in epigenetic regulation. *Nat. Struct. Mol. Biol.* 20 300–307. 10.1038/nsmb.2480 23463315

[B27] Merico D. Isserlin R. Stueker O. Emili A. Bader G. D. (2010). Enrichment map: a network-based method for gene-set enrichment visualization and interpretation. *PLoS One* 5:e13984. 10.1371/journal.pone.0013984 21085593 PMC2981572

[B28] Parveen B. Parveen A. Vohora D. (2019). Biomarkers of osteoporosis: an update. *Endocr. Metab. Immune Disord. Drug Targets* 19 895–912. 10.2174/1871530319666190204165207 30727928

[B29] Pelechano V. Steinmetz L. M. (2013). Gene regulation by antisense transcription. *Nat. Rev. Genet.* 14 880–893. 10.1038/nrg3594 24217315

[B30] Peng S. Cao L. He S. Zhong Y. Ma H. Zhang Y. (2018). An overview of long non-coding RNAs involved in bone regeneration from mesenchymal stem cells. *Stem Cells Int.* 2018:8273648. 10.1155/2018/8273648 29535782 PMC5829309

[B31] Quinn J. J. Chang H. Y. (2016). Unique features of long non-coding RNA biogenesis and function. *Nat. Rev. Genet.* 17 47–62. 10.1038/nrg.2015.10 26666209

[B32] Ritchie M. E. Phipson B. Wu D. Hu Y. Law C. W. Shi W. (2015). limma powers differential expression analyses for RNA-sequencing and microarray studies. *Nucleic Acids Res.* 43:e47. 10.1093/nar/gkv007 25605792 PMC4402510

[B33] Seeliger C. Karpinski K. Haug A. T. Vester H. Schmitt A. Bauer J. S. (2014). Five freely circulating miRNAs and bone tissue miRNAs are associated with osteoporotic fractures. *J. Bone Miner. Res.* 29 1718–1728. 10.1002/jbmr.2175 24431276

[B34] Silva A. M. Moura S. R. Teixeira J. H. Barbosa M. A. Santos S. G. Almeida M. I. (2019). Long non-coding RNAs: a missing link in osteoporosis. *Bone Res.* 7:10. 10.1038/s41413-019-0048-9 30937214 PMC6437190

[B35] Sivaraj K. K. Adams R. H. (2016). Blood vessel formation and function in bone. *Development* 143 2706–2715. 10.1242/dev.136861 27486231

[B36] Sun J. Zhang Z. Bao S. Yan C. Hou P. Wu N. (2020). Identification of tumor immune infiltration-associated lncRNAs for improving prognosis and immunotherapy response of patients with non-small cell lung cancer. *J. Immunother. Cancer* 8:e000110. 10.1136/jitc-2019-000110 32041817 PMC7057423

[B37] Tan J.-X. Li S.-H. Zhang Z.-M. Chen C.-X. Chen W. Tang H. (2019). Identification of hormone binding proteins based on machine learning methods. *Math. Biosci. Eng.* 16 2466–2480. 10.3934/mbe.2019123 31137222

[B38] Tay Y. Rinn J. Pandolfi P. P. (2014). The multilayered complexity of ceRNA crosstalk and competition. *Nature* 505 344–352. 10.1038/nature12986 24429633 PMC4113481

[B39] Toth R. Schiffmann H. Hube-Magg C. Buscheck F. Hoflmayer D. Weidemann S. (2019). Random forest-based modelling to detect biomarkers for prostate cancer progression. *Clin. Epigenetics* 11:148. 10.1186/s13148-019-0736-8 31640781 PMC6805338

[B40] Tu K. N. Lie J. D. Wan C. K. V. Cameron M. Austel A. G. Nguyen J. K. (2018). Osteoporosis: a review of treatment options. *PT* 43 92–104. PMC5768298 29386866

[B41] Ukon Y. Makino T. Kodama J. Tsukazaki H. Tateiwa D. Yoshikawa H. (2019). Molecular-based treatment strategies for osteoporosis: a literature review. *Int. J. Mol. Sci.* 20:E2557. 10.3390/ijms20102557 31137666 PMC6567245

[B42] Yu G. Wang L. Li Y. Ma Z. Li Y. (2013). Identification of drug candidate for osteoporosis by computational bioinformatics analysis of gene expression profile. *Eur. J. Med. Res.* 18:5. 10.1186/2047-783X-18-5 23448234 PMC3599344

[B43] Yu G. Wang L. G. Han Y. He Q. Y. (2012). clusterProfiler: an R package for comparing biological themes among gene clusters. *OMICS* 16 284–287. 10.1089/omi.2011.0118 22455463 PMC3339379

[B44] Zhou M. Diao Z. Yue X. Chen Y. Zhao H. Cheng L. (2016). Construction and analysis of dysregulated lncRNA-associated ceRNA network identified novel lncRNA biomarkers for early diagnosis of human pancreatic cancer. *Oncotarget* 7 56383–56394. 10.18632/oncotarget.10891 27487139 PMC5302921

[B45] Zhou M. Hu L. Zhang Z. Wu N. Sun J. Su J. (2018a). Recurrence-associated long non-coding RNA signature for determining the risk of recurrence in patients with colon cancer. *Mol. Ther. Nucleic Acids* 12 518–529. 10.1016/j.omtn.2018.06.007 30195788 PMC6076224

[B46] Zhou M. Zhang Z. Zhao H. Bao S. Cheng L. Sun J. (2018b). An immune-related Six-lncRNA signature to improve prognosis prediction of glioblastoma multiforme. *Mol. Neurobiol.* 55 3684–3697. 10.1007/s12035-017-0572-9 28527107

[B47] Zhou M. Zhao H. Wang X. Sun J. Su J. (2019). Analysis of long non-coding RNAs highlights region-specific altered expression patterns and diagnostic roles in Alzheimer’s disease. *Brief Bioinform.* 20 598–608. 10.1093/bib/bby021 29672663

